# Addition of new editors for our journal

**DOI:** 10.1016/j.advnut.2023.100147

**Published:** 2023-11-25

I am pleased to announce that *Advances in Nutrition: An International Review Journal* has added several new scientists to its outstanding groups of Associate Editors and Editorial Board members (https://advances.nutrition.org/advnut-ed-board) . Associate Editors are responsible for the primary handling of most submitted manuscripts including reviewer assignments, and, along with the editor-in-chief, making recommendations for final decisions on manuscripts. Editorial Board members advocate for the journal, provide input into policies, and one member of the board will be asked to review most manuscripts sent for peer review.

Our new Associate Editors are Kimberly O. O’Brien, PhD (https://www.human.cornell.edu/people/koo4), Andrew A. Bremer, MD, PhD (https://dpcpsi.nih.gov/onr/staff/Andrew-Bremer), and Lukas Schwingshackl, PhD (https://www.uniklinik-freiburg.de/institut-fuer-evidenz-in-der-medizin/default-90c740b8d5.html). They are highly recognized leaders in the nutritional science field. Dr. O’Brien is a Professor of Human Nutrition in the Division of Nutritional Sciences at Cornell University. Her career has focused on the use of stable isotopes in the evaluation of dietary mineral requirements, especially in women and children. She is a prior recipient of the Norman Kretchmer Memorial Award in Nutrition and Development of the American Society for Nutrition. Dr. Bremer was recently appointed Director of the National Institutes of Health Office of Nutrition Research (ONR), a key position with responsibility for advancing research in nutritional sciences. He is a renowned pediatrician, pediatric endocrinologist, and internal medicine physician with a PhD in pharmacology. He has expertise in diabetes management and clinical trials research in human disease. Dr. Schwingshackl is a professor and research group leader at the Institute for Evidence in Medicine at the Medical University of Freiburg, Freiburg, Germany. He has broad expertise in evidence synthesis of nutrition epidemiological and interventional studies on the metabolic consequence of nutrients, foods, and dietary pattern on human health. He was named a Highly Cited Researcher in 2020, 2021, 2022 and 2023, and recently was awarded the Max Rubner-Prize for his research.

We welcome 4 new editorial board members as well. They are, Magali Rios-Leyvraz, PhD, Independent Consultant, Université of Fribourg in Switzerland, Kelley S. Scanlon, PhD from the Center for Nutrition Policy and Promotion, Food and Nutrition Service, United States Department of Agriculture, Zulfiqar Bhutta, MBBS, PhD https://www.aku.edu/ighd/Pages/home.aspx and https://www.sickkids.ca/en/staff/b/zulfiqar-bhutta/, and Ashley Vargas, PhD from the National Institutes of Environmental Health Sciences. This group brings both a large global geographic range to our editorial board group and diverse expertise in human research, epidemiology, and critical topics such as global malnutrition and climate change.

Beginning in January 2024 *Advances in Nutrition* moves to a monthly publication format and introduces regular editorials related to published articles. We welcome manuscript submissions across the range of nutritional sciences. Please reach out to me directly with questions and concerns about suitability for publication and fees related to article publication in *Advances in Nutrition*, a gold open access journal since January 2023.

